# Encoding of object location in a scrolling display

**DOI:** 10.1177/20416695241281172

**Published:** 2024-09-29

**Authors:** Yumiko Fujii, Hiromi Morita

**Affiliations:** Institute of Library, Information and Media Science, 13121 University of Tsukuba, Tsukuba, Japan; Center for Medical and Nursing Education, 13131 Tokyo Women's Medical University, Shinjuku, Japan; Institute of Library, Information and Media Science, 13121 University of Tsukuba, Tsukuba, Japan

**Keywords:** object encoding, object location, visual information processing, scrolling display, sequential view, visual working memory

## Abstract

There are two main characteristics of visual information processing when viewing an image by scrolling on a small screen: viewing the image sequentially, section by section, owing to the limited visible area, and moving the image to view the desired section of the image. In this study, we investigated the effects of these characteristics on the encoding of object location. The participants were required to observe an image containing 10 objects under three viewing conditions without a time limit and to recall the location of the target object. The viewing conditions were a scrolling condition, a moving-window condition in which a fixed image was viewed by moving the window, and a no-window condition in which the entire image was viewed without a window. The results showed that although the recall accuracy did not differ among the conditions, the observation time increased in the order of scrolling, moving-window, and no-window conditions. These results indicate that in a scrolling view, the object location can be encoded with the same accuracy as that in a full view; however, more time is required for encoding. This finding suggests that viewing the image sequentially and moving the image degrade the encoding of object location.

In our daily lives, we browse various images by scrolling on the small screen of a mobile terminal such as a smartphone or tablet. This type of visual input accounts for most daily visual inputs, given that mobile terminals are widely available. Although several studies (Fujii & Morita, 2020; Sho & Morita, 2023) have investigated the characteristics of such visual inputs, insufficient findings have been accumulated.

Compared with processing in full view, visual information processing in a scrolling display through a small screen has the following two main characteristics: (1) The image is viewed within a limited window, so the entire image cannot be captured simultaneously; therefore, the sections of the image that are viewed sequentially must be integrated into the visual working memory to build up a representation of the entire image. (2) The image is moved to view the desired section, causing shifts in the absolute location of the image during viewing, which can increase the cognitive load on image encoding, especially the encoding of object location in an image.

Previous studies have investigated image perception and memory in a scrolling display, a particular visual environment with the above characteristics of visual information processing. Fujii and Morita (2020) examined the effects of scrolling displays on image perception using a visual search task. They reported that search efficiency was lower in a scrolling view than in a full view. Sho and Morita (2023) examined the effects of scrolling displays on long-term memory for objects in the image. In their experiment, participants observed an image containing multiple objects without a time limit. Immediately after the observation, they were tested on recognition of the objects in the image and then again tested after a 1-h delay. The results suggested that there is no difference in the encoding efficiency into long-term memory in the scrolling view compared to the full view. Given that the absolute location of the image shifts during observation in the scrolling display, the scrolling display may affect the encoding of the object location rather than that of the object itself. However, the effect of scrolling displays on the encoding of object location has not been directly investigated.

Regarding related studies, we discuss the effects of each of the two characteristics of visual information processing in a scrolling display on image perception and memory. First, previous studies have investigated the effect of observation within a limited window area on image perception using the gaze-contingent window method (Bertera & Rayner, 2000; Foulsham et al., 2013; Loschky & McConkie, 2002). For example, Bertera and Rayner (2000) reported that search times in a visual search task increased with decreasing window sizes. However, previous studies using the gaze-contingent window method did not investigate whether objects perceived sequentially within a limited window can be accurately positioned in memory at the original location in the image.

Furthermore, the effects of the viewing method on object-location encoding have been investigated in previous studies by comparing the simultaneous and sequential viewing of multiple objects in a scene. Several studies have reported better performance on memory tasks for object location or the relationship between object locations with simultaneous viewing than with sequential viewing (Blalock & Clegg, 2010; De Rover et al., 2008; Read et al., 2016). For example, Blalock and Clegg (2010) compared the performance of a change-detection task for the spatial configuration of four items under simultaneous and sequential presentation conditions. Their experiment required the participants to report that the configuration of items was “different” between the sample and test arrays if all items moved to a new location or if two random items were swapped and that it was “same” if the items moved in parallel while maintaining the configurations between them or if all items were in the same location. The results showed that the task accuracy decreased with sequential presentations. Moreover, this decrease was particularly significant when items were swapped, suggesting that sequential presentation impairs the memory of item-level location information. However, in the sequential presentation conditions of these previous studies, the objects were presented in a predetermined order for a fixed duration. In contrast, in a scrolling display, sections of the image are presented in an exploratory manner by allowing the viewer to control the order and duration of the object presentation. The scrolling display may have had a different effect on object location encoding than the sequential presentation condition used in previous studies due to this difference in viewing control.

Second, the effects of shifts in the absolute location of an image during viewing have not yet been thoroughly investigated. However, it has been proposed that an object is encoded in memory based on its location (Kahneman et al., 1992). Furthermore, previous studies have suggested that location plays a central role not only in encoding but also in retention and retrieval (Hollingworth, 2007; Treisman & Zhang, 2006). In addition, Hollingworth and Rasmussen (2010) suggested that when an object or its frame moves, the memory of the relationship between the object and its location is updated in association with the new location if the motion can be perceived. In light of these previous studies, it is conceivable that shifts in image location during viewing in a scrolling display may not significantly degrade the recall performance for the object location but may at least impose some cognitive load. However, when we encode objects in an image displayed on a scrolling display, each object is encoded in memory based on its location within the screen because the object is presented within the screen. An interesting question is whether an object that extends beyond the screen by subsequent scrolling can be retained in visual working memory with its original position in reference to the image. It is interesting to understand whether the invisible object is retained in its original position in the image while scrolling, and if so, what source of information can recover its original position in the image.

In summary, there are two characteristics of visual information processing in a scrolling display through a small screen: viewing the image within a limited window and shifting the absolute location of the image during viewing. However, it is unclear how these characteristics affect the encoding of the location of an object in an image. Therefore, the purpose of the present study was to clarify the effect of two characteristics of visual information processing in a scrolling display on the encoding of object location. We believe that the present study can clarify the effect of viewing the image by scrolling within a small screen on image cognition, and the results will not only be useful for practical applications but may also have implications for previous vision research.

For this purpose, we conducted an experiment in which participants were required to observe a sample image of 10 randomly placed objects presented on a touchscreen display and then recall the location of the target object in the sample image. Three viewing conditions were set: scrolling, moving-window, and no-window (Fujii & Morita, 2020). In the scrolling condition, the image was pulled into a small fixed window for viewing by scrolling the screen. In contrast, in the moving-window condition, a small window was moved to the desired section of the fixed image for viewing. In both conditions, image sections were presented sequentially in the window; however, in the moving-window condition, unlike in the scrolling condition, the absolute location of the image did not shift during viewing. Therefore, a comparison between the scrolling and moving-window conditions allowed us to reveal the effect of shifts in the absolute location of the image. Under the no-window condition, the entire image was viewed without a window. A comparison between the no-window condition and other conditions revealed the effect of the sequential presentation of image sections.

Fujii and Morita (2020), who examined the characteristics of visual search in scrolling displays, categorized image scanning traces into movement and pause states. They revealed that frequent pauses were interjected with fast image shifts over short distances under the scrolling display. In contrast, slow window movements over continuous distances were interjected with a few pauses under the moving-window display, suggesting that this difference in dynamic properties may have caused the difference in search efficiency. The present study examined whether the dynamic properties of image scanning in image observation for the object-location recall task reflected the method of encoding the object location in the image during scrolling.

## Methods

### Participants

A total of 22 undergraduate and graduate students (12 males and 10 females; age range, 19–26 years; mean age 21.3 years; all right-handed) with normal or corrected-to-normal vision were included in the study. Each participant received a full explanation of the experiment and provided written informed consent. The present study was approved by the Ethics Committee of the Institute of Library, Information, and Media Science at the University of Tsukuba (approval no. 20–125). It was conducted following the Code of Ethics and Conduct of the Japanese Psychological Association.

### Apparatus

Visual stimuli were presented on a 23-inch touchscreen liquid crystal display (EIZO Inc., FlexScan T2381 W, 1920 × 1080 pixel resolution) controlled by a computer operating on Microsoft Windows 10 (Dell Inc., Dell Precision T1650), MATLAB (Mathworks Inc.), and the Psychophysics Toolbox (Brainard, 1997; Kleiner et al., 2007; Pelli, 1997).

The chair height and display angle were adjusted so that seated participants could easily operate the screen. A chin rest was not used because it would have fixed the participant's head, making the touch operation inconvenient. Consequently, the viewing distance differed among the participants. When the visual angle was calculated, the viewing distance was estimated to be 45 cm. The experiments were conducted under normal lighting conditions.

### Stimulus

The 180 object photos selected from the Bank of Standardized Stimuli (Brodeur et al., 2010; Brodeur et al., 2014) were divided into six object sets of 30 photos each, and these sets were assigned to a practice block and five experimental blocks. Each experimental block was run under different conditions. The assignment of object sets to the conditions was counterbalanced across participants. For each trial in a block, 10 objects were randomly selected from an object set of 30 photographs and arranged in the image.

**Figure 1.**
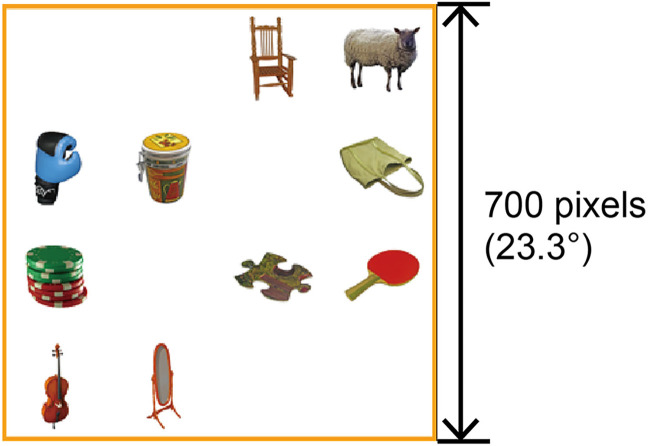
An example of the stimulus image.

As shown in Figure 1, the stimulus images were created by dividing a 700 × 700-pixels white background area (visual angle of 23.3 × 23.3°) into equal 4 × 4 virtual grids, that is, 16 squares, of which 10 squares were occupied by objects. We created 180 different configurations of 10 squares by randomly arranging them in a 4 × 4 square array in advance. These configurations were divided into 18 sets, of which six were randomly selected for each participant. One set was assigned to a practice block, and the remaining sets were assigned to each experimental block.

The target object and its position in the object location recall task were randomly chosen to be unique within the block. An orange inner frame for the background (line width of 7 pixels) was presented so that the participants could easily find the edge of the stimulus image.

### Procedure

**Figure 2.**
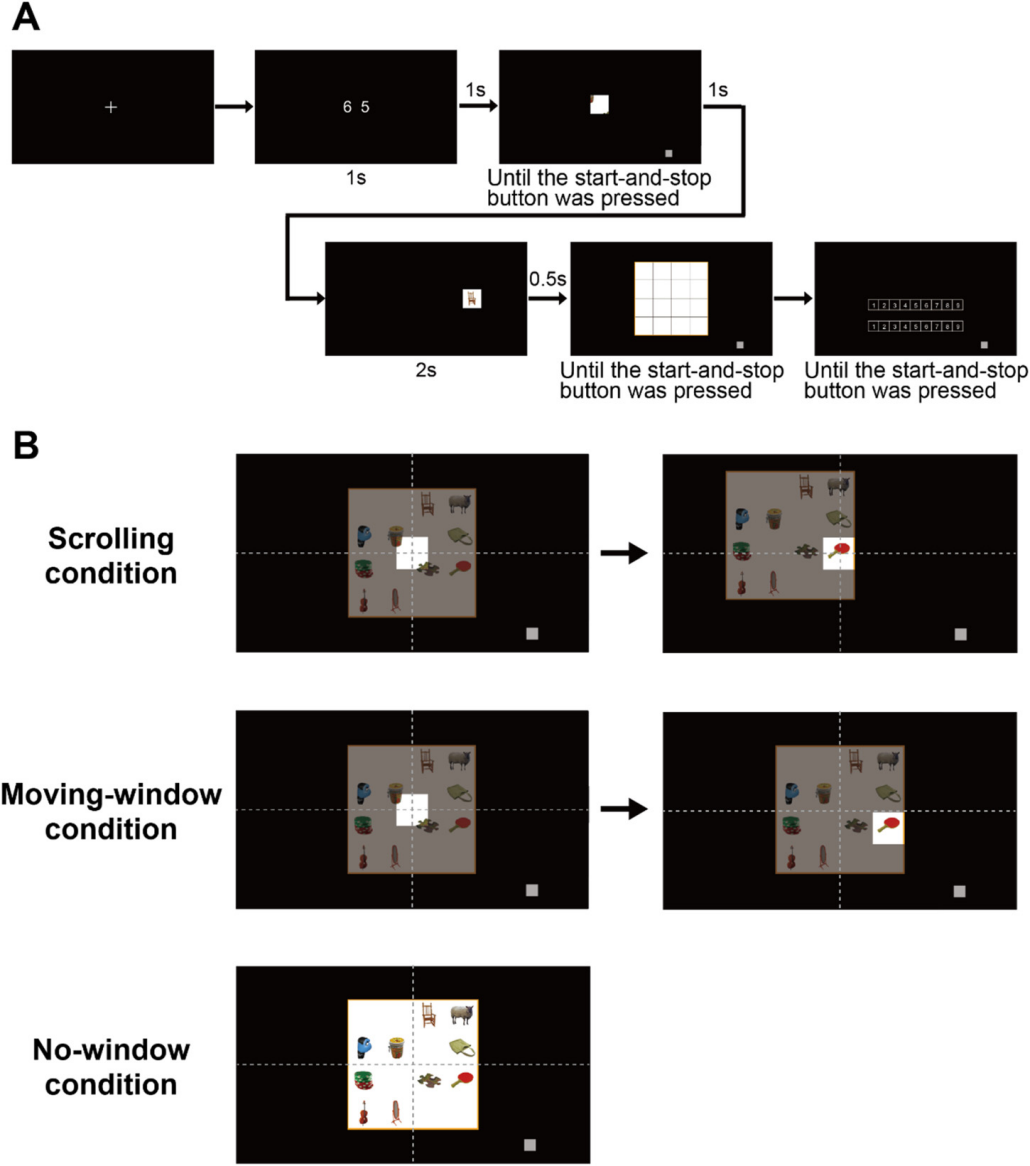
Schematic illustration of the experimental procedure. (A) The sequence of a trial. (B) Positional relationship between the stimulus image and the small window for the three viewing conditions. In the scrolling condition, the image is moved, and the window is fixed, whereas in the moving-window condition, the image is fixed, and the window is moved.

Note: Although the mask is gray and transparent in this figure, it was black in the actual experiment to mask the stimulus image. The gray dotted lines drawn in the horizontal and vertical center of the display were not displayed in the actual experiment.

The experimental procedure is illustrated in Figure 2A. We conducted an object location recall task. The trial started by touching a white fixation cross that appeared at the center of the screen. A random two-digit number was then presented at the center of the screen for 1 s. The participants were required to repeat this number verbally until the end of the trial to interfere with the verbal encoding of the subsequently presented image. After a 1-s blank interval, the stimulus image was presented. The participants were required to observe the stimulus image under the three viewing conditions (Figure 2B) and memorize the location of each object in the image.

In the no-window condition, the participants viewed the entire image displayed at the center of the screen. For the scrolling and moving-window conditions, the participants viewed the stimulus image through a 175 × 175-pixels window (referred to as the small window, with a visual angle of 5.9 × 5.9°) or a 233 × 233-pixels window (referred to as the large window, with a visual angle of 7.9 × 7.9°)^1^. The initial positions of both the window and stimulus image were at the center of the screen. In the scrolling condition, participants moved the image by touching and sliding their index finger within the window to pull the desired section of the image into the fixed central window. Even if their fingers exited the window boundaries, the image could follow them as long as they touched the image area. In contrast, in the moving-window condition, the participants moved the window by touching and sliding their index finger within the window to display the desired section of the fixed image. That is, in the scrolling condition, the image is moved, and the window is fixed, whereas in the moving-window condition, the image is fixed, and the window is moved. We set two window size conditions to examine whether the effect of viewing in a scrolling display on object-location encoding depends on the size of the visible window area. Given that a previous study using the gaze-contingent window method reported that visual search performance decreases with decreasing window size (Bertera & Rayner, 2000), we expected the effect of the scrolling display to be more pronounced at smaller window sizes.

When the participants felt that they had observed the stimulus image sufficiently, they were required to press the gray start-and-stop button (70 × 70 pixels, 2.4 × 2.4° visual angle) at the bottom right of the screen. No time limit was set for these observations because we intended to use the index of observation time to analyze the ease of encoding, including not only the recall accuracy but also the efficiency of image scanning. Additionally, the stimulus images should be viewed freely with fewer restrictions to analyze the dynamic properties of the scan. The observation time was measured from the presentation of the stimulus image until the start-and-stop button was pressed. After the observation, a 1-s blank interval was followed by the presentation of one randomly selected object from the stimulus image (i.e., the target object) on the left or right side of the screen for 2 s. After a 0.5-s blank interval, the response screen with 4 × 4 grids in the empty image area was displayed. The participants were required to select where the target object was located in the stimulus image by touching the square. When the participants touched a square, it was colored orange. Participants could change their selection until they pressed the start-and-stop button.

After determining the location of the target object, two sequences from 1 to 9 were displayed at the bottom of the screen. The participants answered the upper first and second digits of the two-digit number presented at the beginning of the trial by touching and selecting numbers from the top and bottom rows of the sequences at the bottom of the screen, respectively. When a number was touched, the number and its surrounding frame turned orange, and the two-digit number touched appeared at the top of the screen. Participants could change their selection of numbers until the start-and-stop button was pressed. All the touch operations were performed using the index finger of the dominant hand.

### Design

Three viewing conditions were considered: scrolling, moving-window, and no-window. In addition, there were two window sizes for the scrolling and moving-window conditions: small and large. Thus, for each of the five conditions (scrolling/small, scrolling/large, moving-window/small, moving-window/large, and no-window), a practice block of two trials and an experimental block of ten trials were conducted. The order of the conditions was counterbalanced among participants.

### Analysis of the Dynamic Properties of the Scan

We analyzed the coordinates of the center position of the window in relation to the stimulus image for the scrolling and moving-window conditions to examine the dynamic properties of the scan. The sampling rate was 60 Hz. When the participants removed their fingers from the screen so that neither the stimulus nor the window moved, no samples were recorded. It was possible to determine when and for how long participants took their fingers off the screen because sample timestamps were recorded.

Based on a study by Fujii and Morita (2020), we categorized each period between consecutive samples into movements or pauses using the following procedure: (1) If the rate of movement of the central window position in a period exceeded 60 pixels per second (2.0°/s), the period was categorized as movement; otherwise, it was categorized as a pause. (2) A movement series in which the total distance was less than 58.25 pixels (a quarter of the large window width; visual angle of 1.9°) was re-categorized as a pause. (3) A pause series in which the total time was less than 200 ms was re-categorized as movement. We calculated the number of pauses, mean duration of a pause, cumulative duration of pauses, mean distance of movement, cumulative distance of movements, and mean speed of movement per trial. The cumulative duration of pauses was calculated by summing the durations of all pauses on each trial. The cumulative distance of movements was calculated similarly.

### Classification of Pause Locations in Image Scanning

**Figure 3.**
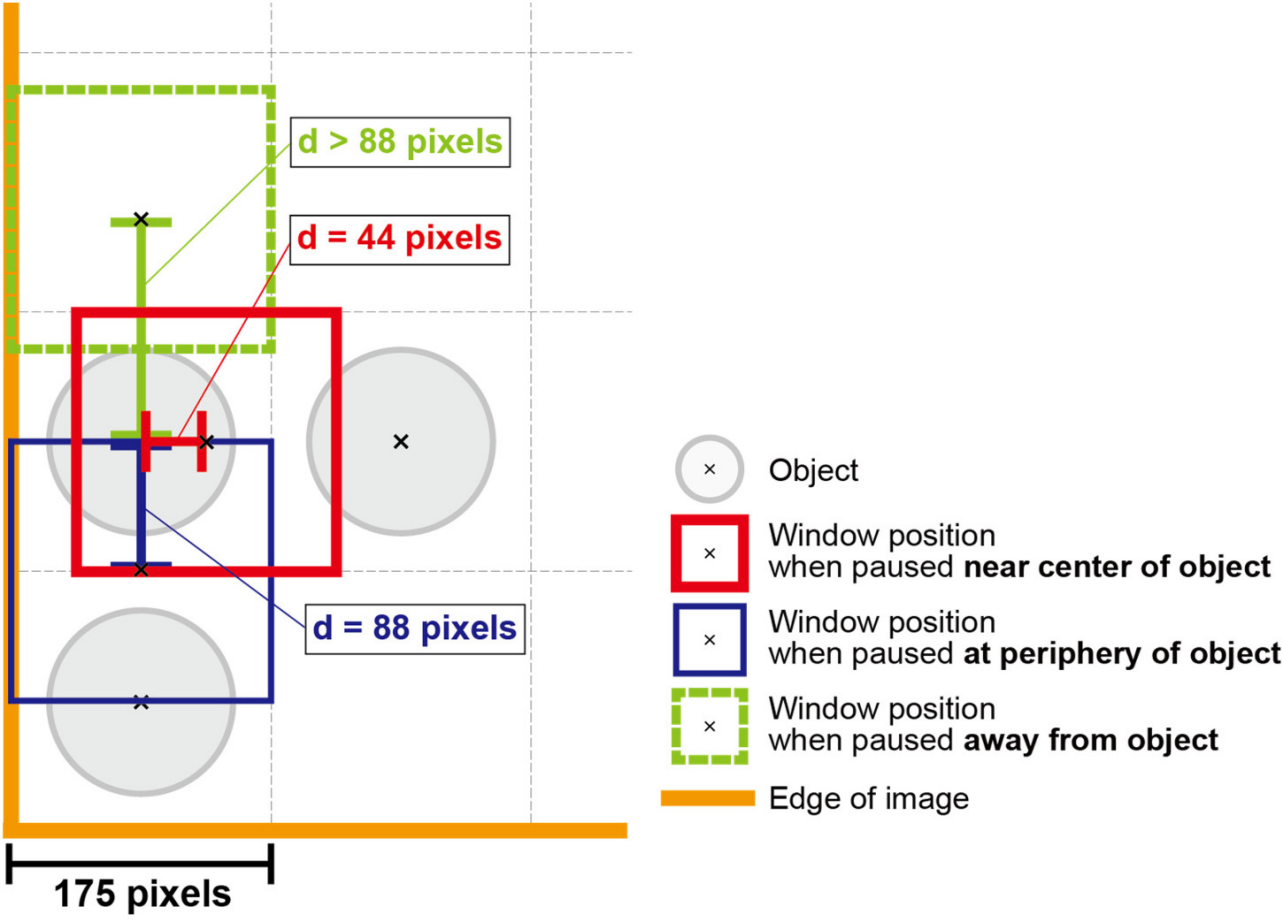
Illustration of classification of pause locations. The “d” indicates the distance from the pause point to the nearest object. If “d” is 44 pixels or less, the pause point was defined as being near the center of the object (The red bold box indicates an example of window position in this case.); if “d” is greater than 44 pixels and less than or equal to 88 pixels, the pause point was defined as being in the surroundings of the object (The blue thin box indicates an example of the window position in this case.); if “d” is greater than 88 pixels, the pause point was defined as being away from the object (The green dotted box indicates an example of the window position in this case.).

For the small window condition in the scrolling and moving-window conditions, we calculated the distance from the pause location to the center of the nearest object. Figure 3 illustrates the classification of pause locations. All pauses were classified as a pause near the center of the object, at the periphery of the object, or away from the object, according to the following criteria. A pause point was defined as being near the center of the object if the distance from the pause point to the nearest object was 44 pixels or less (i.e., approximately one-quarter of one side of a square in a 4 × 4 virtual grid on which the object could be placed). In this case, only one object mainly occupied the window area. A pause point was defined as being in the surroundings of the object if the distance from the pause point to the nearest object was greater than 44 pixels and less than or equal to 88 pixels. In this case, another object could appear in the window. It was likely that no object was presented as a whole if the distance from the pause point to the nearest object was greater than 88 pixels; this case was defined as a pause away from the object. If the location of each object is memorized independently of the surrounding objects, the window is expected to pause at the center of the object. However, if the locations of objects are memorized in relation to each other, then pauses with multiple objects in the window would occur because it is beneficial for memorizing the relation of location between objects. This pause location analysis was performed only for the small window condition and not for the large window condition because the objects placed in the corner of the image could not be presented in the center of the large window.

### Statistical Analysis

Data from two participants (both male) whose mean observation time or correct response rate for all conditions was more than three standard deviations away from the mean for all participants were excluded from the analyses and considered as outliers. Thus, we analyzed the data of 20 participants. To examine whether the effects of the characteristics of visual information processing in the scrolling display on the encoding of the object location became more pronounced with window size, we conducted a two-factor (two viewing conditions × two window sizes) analysis of variance (ANOVA) on the observation time and correct response rate for the scrolling and moving-window conditions in which images were observed within the limited window. However, no interaction was observed between the viewing condition and window size in these ANOVAs, indicating that the effect of the characteristics of visual information processing in the scrolling display on the encoding of the object location did not vary with window size. Therefore, for the scrolling and moving-window conditions, we collapsed the window size factor and averaged the data for both window sizes.

Observation times and correct response rates were analyzed using one-way repeated measures ANOVAs with viewing condition (scrolling, moving-window, versus no-window) as the factor. In the analysis of the dynamic properties of the scan, the numbers of pauses, mean durations of a pause, cumulative durations of pauses, mean distances of movement, cumulative distances of movements, and mean speeds of movement per trial were compared between the scrolling and moving-window conditions via a paired t-tests. Furthermore, in the analysis of the pause location, the numbers of pauses were analyzed using a two-way repeated measures ANOVA with viewing condition (scrolling versus moving-window) and pause location (center of the object, the periphery of the object, versus away from the object) as factors.

## Results

### Task Performance

#### Observation Time

**Figure 4.**
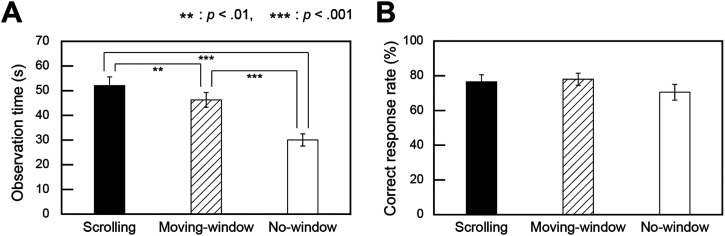
Task performance. (A) Observation time, (B) Correct response rate. Error bars indicate standard errors.

Figure 4A shows the mean observation time for each viewing condition. We compared the no-window condition and the means for the two window sizes in the scrolling and moving-window conditions to examine the differences in observation time between viewing conditions. A one-way repeated measures ANOVA (three viewing conditions: scrolling, moving-window, versus no-window) revealed that the main effect was significant (*F*(2, 38) = 60.1, *p *< .001, η_p_^2 ^= 0.8). Post-hoc analyses with Bonferroni-Holm corrections showed significant differences among all conditions, with the scrolling, moving-window, and no-window conditions having the longest observation times in that order (scrolling versus no-window: *p *< .001, *d *= 1.6; moving-window versus no-window: *p *< .001, *d *= 1.3; scrolling versus moving-window: *p *< .01, *d *= 0.4).

#### Correct Response Rate

Figure 4B shows the mean correct response rate for each viewing condition. Statistical analysis of the correct response rate was performed in the same manner as that for the observation time. A one-way repeated measures ANOVA (three viewing conditions: scrolling, moving-window, versus no-window) with a Greenhouse–Geisser correction revealed no significant main effect (*F*(1.5, 29.0) = 2.1, *p *= .2, η_p_^2 ^= 0.1). These results implied that the correct response rates for the three viewing conditions did not differ.

### Dynamic Properties of Scan

#### Scan Path of One Participant

**Figure 5.**
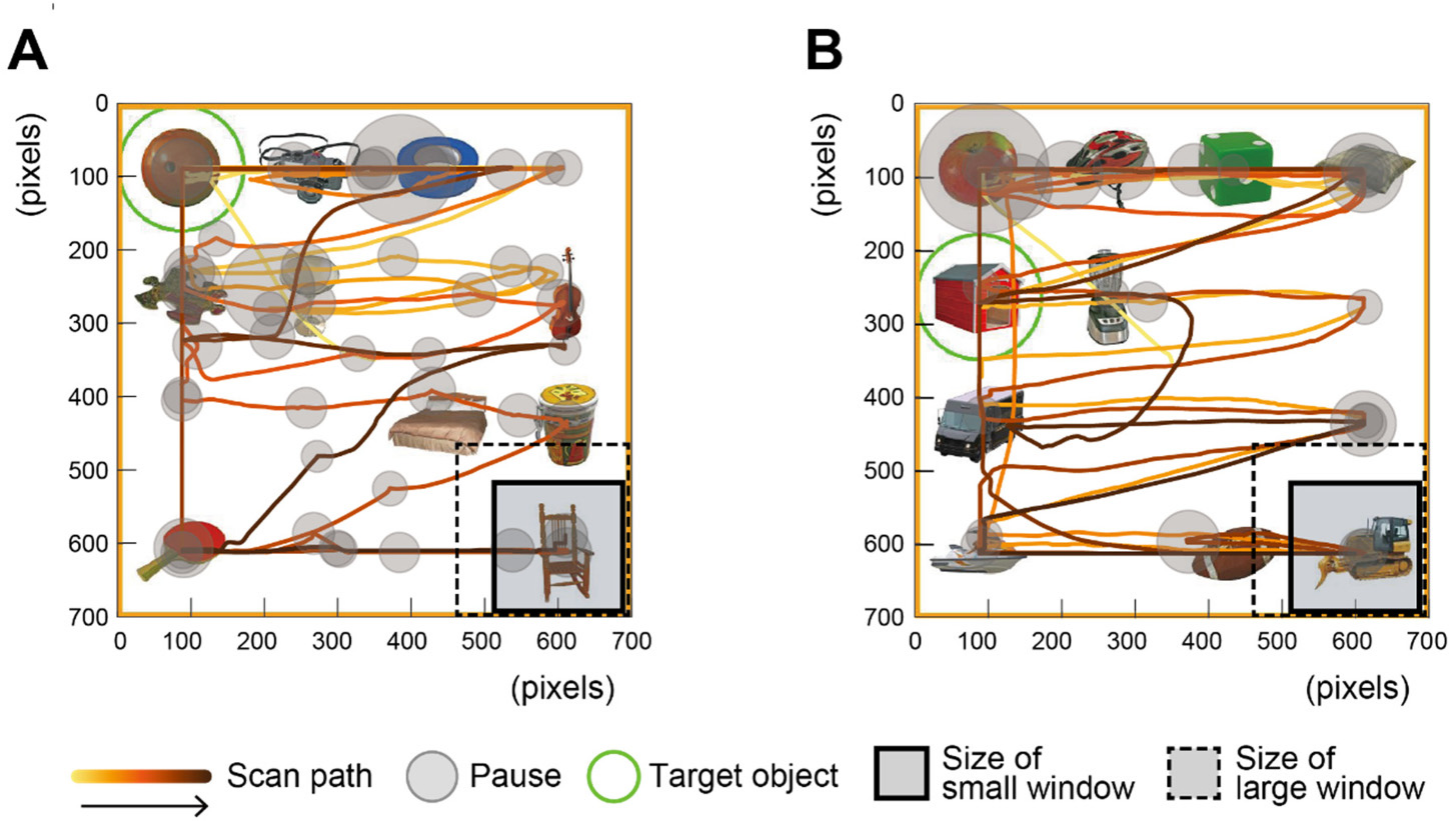
Trace of the central position of the window in relation to the stimulus image. The gradient line starting in yellow and ending in brown indicates the order of the scan path (i.e., the path starting in yellow and ending in brown). (A) The trace for the correct trial in the scrolling condition from one of the participants; (B) The trace for the correct trial in the moving-window condition from the same participant.

Figure 5 shows the scan path for the correct trial in the scrolling (small window) and moving-window (small window) conditions of the same participant. These scan paths indicate that the image was scanned with more frequent pauses in the scrolling condition than in the moving-window condition.

#### Number of Pauses and Pause Duration

**Figure 6.**
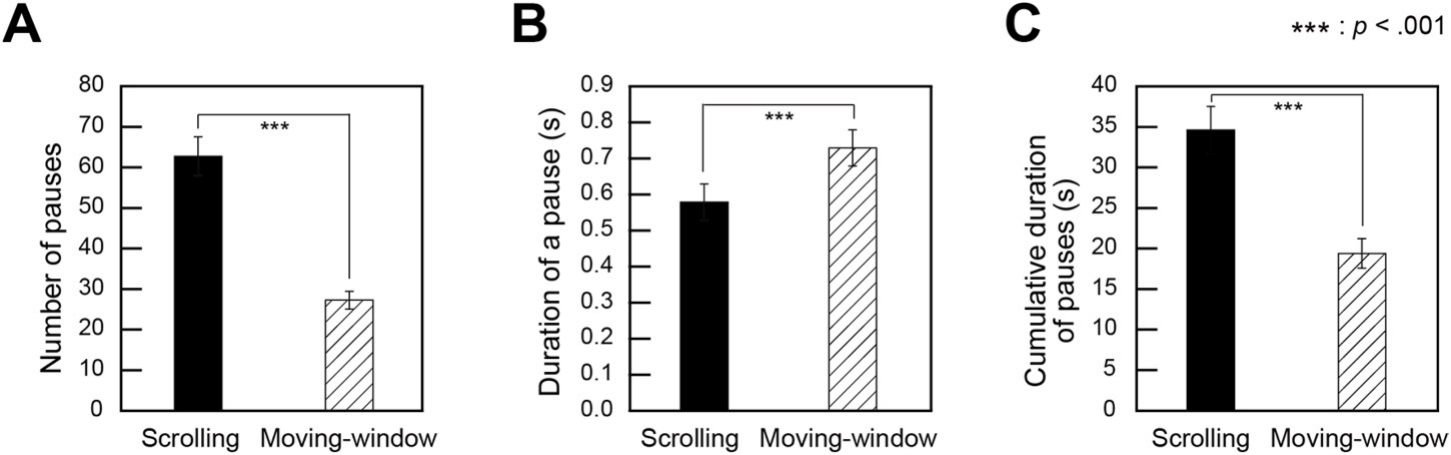
Properties of pauses in image scanning. (A) The number of pauses, (B) The mean pause duration, (C) The mean cumulative duration of pauses. Error bars indicate standard errors.

The number of pauses and the mean duration of a pause were calculated per trial for the scrolling and moving-window conditions. We compared the means of the two window sizes in the scrolling and moving-window conditions to examine the differences in these measures between the viewing conditions. Figure 6 shows the mean number of pauses, mean pause duration, and mean cumulative duration of pauses for the scrolling and moving-window conditions.

Paired *t*-tests were conducted on the number of pauses with the viewing condition (scrolling versus moving-window) as a factor, and the results revealed that the number of pauses was significantly greater in the scrolling condition than in the moving-window condition (*t*(19) = 9.4, *p *< .001, *d *= 2.1).

The same paired *t*-test on pause duration revealed that the duration was significantly shorter for the scrolling condition than for the moving-window condition (*t*(19) = −4.2, *p *< .001, *d *= 0.7).

The same paired *t*-test on the cumulative duration of pauses revealed that the cumulative duration was significantly longer for the scrolling condition than for the moving-window condition (*t*(19) = 8.2, *p *< .001, *d *= 1.4).

#### Distance and Speed of Movement

**Figure 7.**
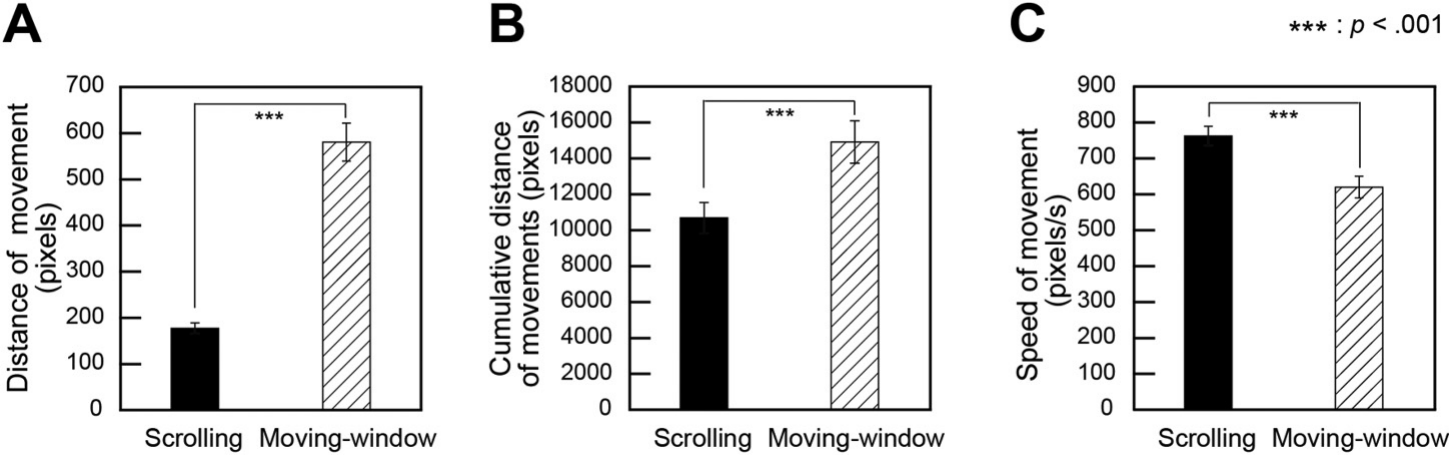
Properties of movement in image scanning. (A) The mean distance of movement, (B) The mean cumulative distance of movements, (C) The mean speed of movement. Error bars indicate standard errors.

The mean distance and speed of movement were calculated per trial for the scrolling and moving-window conditions. The same statistical analyses used for the pause data were also applied to the movement data. Figure 7A–C show the mean distance of movement, mean cumulative distance of movements, and mean speed of movement for the scrolling and moving-window conditions, respectively.

A paired *t*-test was conducted on the distance of movement with the viewing condition (scrolling versus moving-window) as a factor, revealing that the distance of movement was significantly shorter for the scrolling condition than for the moving-window condition (*t*(19) = −10.3, *p *< .001, *d *= 3.0).

The same paired *t*-test on the cumulative distance of movements revealed that the cumulative distance was significantly shorter for the scrolling condition than for the moving-window condition (*t*(19) = −6.9, *p *< .001, *d *= 0.9).

The same paired *t*-test on the speed of movement revealed that the speed was significantly faster for the scrolling condition than for the moving-window condition (*t*(19) = 6.1, *p *< .001, *d *= 1.1).

#### Pause Location

**Figure 8.**
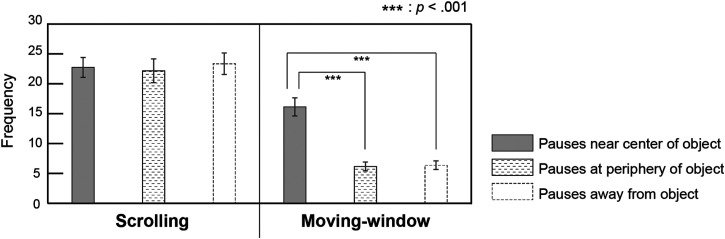
The mean number of pauses near the center of the object, at the periphery of the object, and away from the object. Error bars indicate standard errors.

Figure 8 shows the mean number of pauses near the center of the object, at the periphery of the object, and away from the object. We compared the numbers of these three pause locations to examine how the image was viewed during the pause. For the number of pauses, a two-way repeated measures ANOVA (two viewing conditions: scrolling versus moving-window × three pause locations: center of the object, the periphery of the object, versus away from the object) with a Greenhouse–Geisser correction revealed significant main effects of viewing condition (*F*(1, 19) = 121.3, *p *< .001, η_p_^2 ^= 0.9) and pause location (*F*(1.5, 28.9) = 19.0, *p *< .001, η_p_^2 ^= 0.5). Moreover, there was a significant interaction between the viewing condition and pause location (*F*(1.2, 22.1) = 19.5, *p *< .001, η_p_^2 ^= 0.5). For the interaction between the viewing condition and pause location, separate ANOVAs for each condition revealed that the simple main effect of pause location was significant for the moving-window condition (*F*(1.4, 27.4) = 53.0, *p *< .001, η_p_^2 ^= 0.7), but not for the scrolling condition (*F*(1.3, 24.8) = 0.3, *p *= .6, η_p_^2 ^= 0.02). Further post-hoc analyses with Bonferroni corrections revealed that, for the moving-window condition, the mean number of pauses was greater near the center of the object than those at the periphery of the object (*p *< .001, *d *= 1.9) and away from the object (*p *< .001, *d *= 1.8). This finding implies that the number of pauses did not differ between the three positions in the scrolling condition, whereas in the moving-window condition, pauses near the center of the object were more frequent than those at the periphery of the object or away from the object.

## Discussion

Visual information processing in a scrolling display through a small screen of a mobile terminal has two main characteristics: viewing the image within a limited window and shifting the absolute location of the image during viewing. The present study aimed to clarify how these two characteristics of visual information processing in a scrolling display affect the encoding of the object location.

The results of the object-location recall task showed that although the correct response rate did not differ between the viewing conditions, the time required to observe the image increased in the order of the scrolling, moving-window, and no-window conditions. These results indicate that when there is no time limit for viewing, the scrolling view requires more time to encode the object's location than the full view, even though the object's location can be encoded with the same accuracy. This finding is consistent with previous studies reporting that when an image is viewed sequentially in small sections, the content of the image can be accurately perceived if it is observed for a sufficiently long time (Saida & Ikeda, 1979). However, previous studies have mainly examined whether the content of a large image can be encoded and have not examined whether the location of individual objects in the image is encoded accurately, as we examined in the present study. A further difference between the previous studies and the scrolling view in the present study was that in the scrolling condition of the present study, the section of the image was viewed within a fixed screen by moving the image, whereas in previous studies, each section of the image was viewed at the original location of the section, as in the moving-window condition in the present study. Comparing the scrolling and moving-window conditions showed longer observation time in the scrolling condition. Both conditions involve presenting the image within a limited window. However, in the scrolling condition, the image moves while the window remains fixed, whereas in the moving-window condition, the image is fixed while the window moves. This result indicates that encoding object locations may require more time due to the absolute shift in image location during observation.

Studies have reported that memory accuracy for the location of an object in a scene with multiple objects is lower in the sequential presentation condition than in the simultaneous presentation condition (Blalock & Clegg, 2010; De Rover et al., 2008; Read et al., 2016). Additionally, Bharti et al. (2020) performed a change-detection task for color and shape in which an object in a multi-object array was presented sequentially for 250 ms each. The results showed that object location information, which was advantageous for the task when all objects were presented simultaneously, was not advantageous when the objects were presented sequentially. These studies suggest that objects may not be sufficiently associated with their location in visual working memory when presented sequentially for short durations. In studies where the presentation time of the stimuli was limited, sequential presentation affected accuracy. In contrast, in the present study, the accuracy was the same but the observation time was longer for the scrolling condition than for the no-window condition because participants could observe the image for as long as they wanted.

Regarding the encoding of the content of an image while moving it, Hollingworth and Rasmussen (2010) suggested that if a clear perception that an object has moved is obtained, the features of that object in the memory can be appropriately associated with the new location to which it has moved. In the present study, the accuracy did not differ between the scrolling and moving-window conditions, indicating that the participants could recognize the object location accurately enough, even when the image moved during viewing. As one possibility, this finding suggests that the participants might recognize the movement of the objects and update their locations in memory when they were not visible. However, encoding object location took longer in the scrolling condition than in the moving-window condition despite similar recall accuracy. The longer encoding time suggests that more cognitive load might be imposed in the scrolling condition. Further research will be conducted to determine how the movement of an image during viewing affects working memory for non-displayed objects within the window.

We analyzed the dynamic properties of image scanning to determine why observation time increased under scrolling conditions. Consistent with those of a previous study (Fujii & Morita, 2020), our results showed that frequent pauses were interjected with fast image shifts over short distances in the scrolling condition, whereas slow window movements over continuous distances were interjected with a few pauses in the moving-window condition. Furthermore, in the scrolling condition, the cumulative duration of pauses was longer, and the cumulative distance of movements was shorter than that in the moving-window condition. These results suggest that the longer observation time in the scrolling condition was due to the accumulation of frequent pauses required for image observation but not to the accumulation of the distance of movement.

The reason participants frequently paused in the scrolling condition may relate to the hypothesis that an object is encoded in visual working memory based on its location (Kahneman et al., 1992). In the scrolling display, the desired object is displayed on the screen and encoded in visual working memory based on its location. The same object extends beyond the screen when the image is moved and another object is displayed. Hence, the object's location in relation to the screen shifts while the object is invisible. Therefore, it might be necessary for the observer to frequently pause to recalculate the location of the invisible object based on image movement. On the other hand, because the desired object is displayed and encoded at its location in the moving-window condition, it is not necessary to update the location, regardless of how long the user moves the window.

Furthermore, we investigated the locations of frequent pauses in the scrolling condition. We assumed that when attempting to encode an object's location, the observers would display it around the center of the screen to focus their attention on it. In contrast, pauses at the periphery of an object are sometimes beneficial for encoding the relative locations between objects because these pauses enable neighboring objects to be displayed on the screen simultaneously. Therefore, we classified the pauses into three types: “near the center of the object,” “at the periphery of the object,” and “away from the object.” The results showed that in the moving-window condition, pauses near the center of the object occurred more frequently than the other types of pauses, whereas, in the scrolling condition, the number of pauses was the same for all three types. As mentioned above, observers frequently paused while scrolling, presumably to recalculate the locations of objects extending beyond the window. It is, therefore, consistent that not only pauses near the center of the object but also pauses at its periphery and away from it occur with equal frequency. In addition, the fact that pauses in the periphery occurred as frequently as pauses near the center of the object implies that multiple objects were presented simultaneously on some occasions, which may have helped participants perceive the relative location of objects. Perception of the relative location of the objects may have compensated for the instability of the object's location in relation to the screen. Future research is needed to clarify the role of the relative location of objects in the encoding and memory retention of object locations in scrolling displays.

In a previous study that investigated the effects of a scrolling display on image perception using a visual search task, results similar to those of the present study were reported for the dynamic properties of image scanning, except for the following result (Fujii & Morita, 2020). In contrast to the previous study by Fujii and Morita (2020), the duration of the pause was shorter in the scrolling condition than in the moving-window condition in the present study. Such a difference could have been influenced not only by the type of experimental task but also by the experimental conditions (e.g., image size, window size, and/or stimulus content). Even with these small differences, the dynamic properties of image scanning in the object-location recall task were similar to those in the visual search task. Further studies are required to determine whether the dynamic properties of image scanning in a scrolling display are observed in other task situations.

The present study has some limitations. First, the longer observation time under the viewing condition in which participants observed through a limited window (i.e., the scrolling and moving-window conditions) compared to the no-window condition might be due not only to problems in encoding but also to the inclusion of hand motions during scrolling operations in order to observe the image. The inclusion of hand motions could increase the task complexity for participants. Second, because the present experiment was a recall task, the encoding time include the search and scanning time to encode individual object locations. Third, the increased time required to encode the object location in the scrolling condition than in the moving-window condition may be attributed not only to the shift in the absolute location of the image during viewing but also to the difference in operability. The direction of the gaze that captures the object and the direction of the window or image movement coincide in the moving-window condition but not in the scrolling condition. Therefore, the scrolling operation may impose a greater cognitive load on the scrolling condition. Future studies should consider visual information processing in scrolling displays by focusing on this aspect of scrolling operability.

Nonetheless, although the previous studies examined the efficiency of visual search (Fujii & Morita, 2020) and the recognition of objects (Sho & Morita, 2023) in scrolling displays, no study has examined the encoding of object location in a scrolling display. The present study is significant because the results revealed that it takes time to accurately encode the location of objects in an image when the image is viewed on a scrolling display. While previous studies suggested that the characteristics of visual information processing in a scrolling display do not affect the long-term encoding of the object, the present study suggests that they do affect the short-term encoding of the object's location. Moreover, the reason for this was interpreted in the present study within the framework of cognitive psychology.

In conclusion, the present study revealed that even with the scrolling display, the object location can be encoded with some accuracy. However, it takes more time to encode than it does with a full view. Further results suggested that the longer encoding time was caused by viewing the image sequentially owing to the limited visible area and by shifting the absolute location of the image during viewing. These findings may not only be useful for practical applications but may also provide insights into the mechanisms underlying object location encoding. Specifically, a practical implication is that when using scrolling displays, which demand more time for object location encoding, these limitations should be considered. This issue is also pertinent in video creation, such as social media platforms with scrolling, where camera panning speed and timing should be adjusted to accommodate the viewer's encoding time for object locations. The insight into the mechanisms underlying object location encoding is that even under the constraint of sequentially observing a moving spatial array of objects within a limited visible area, these objects’ locations could be accurately encoded if sufficient time is allowed for the encoding. Furthermore, under such constraint, the location of an object in the array may be encoded using the relative locations of the objects as cues.

## Notes

1. Although smartphones or tablets typically have a rectangular screen shape, this experiment utilized a square window with a 1:1 aspect ratio. The study focused not on exploring the cognitive characteristics of scrolled images on these devices but on investigating how two aspects of visual information processing in scrolling displays affect the encoding of object location in images. Therefore, we opted for a square window, the simplest shape.
